# Two plasmid modules for introducing the auxin-inducible degron into the fission yeast *Schizosaccharomyces pombe* by PCR-based gene targeting

**DOI:** 10.17912/micropub.biology.000442

**Published:** 2021-08-18

**Authors:** Xiuyi Song, Ruoming Xu, Tomoyasu Sugiyama

**Affiliations:** 1 School of Life Science and Technology, ShanghaiTech University

## Abstract

Targeted protein degradation is a powerful approach to study and inhibit protein function *in vivo*. Introduction of the auxin-inducible degron (AID) system to the fission yeast *Schizosaccharomyces pombe* was previously reported, but, to the best of our knowledge, no plasmid for constructing AID-tagged fission yeast strains has been described so far. Here, we describe two plasmids that facilitate the introduction of the mini auxin-inducible degron (mAID) tag with a FLAG epitope or GFP by the conventional PCR-based gene targeting method. Our experimental verification indicated that PCR-based mAID tagging is straightforward and that the auxin-degron system is useful for studying essential proteins in *S. pombe*.

**Figure 1. Verification of two mini auxin-inducible degron (mAID) tagging plasmids for Schizosaccharomyces pombe:**
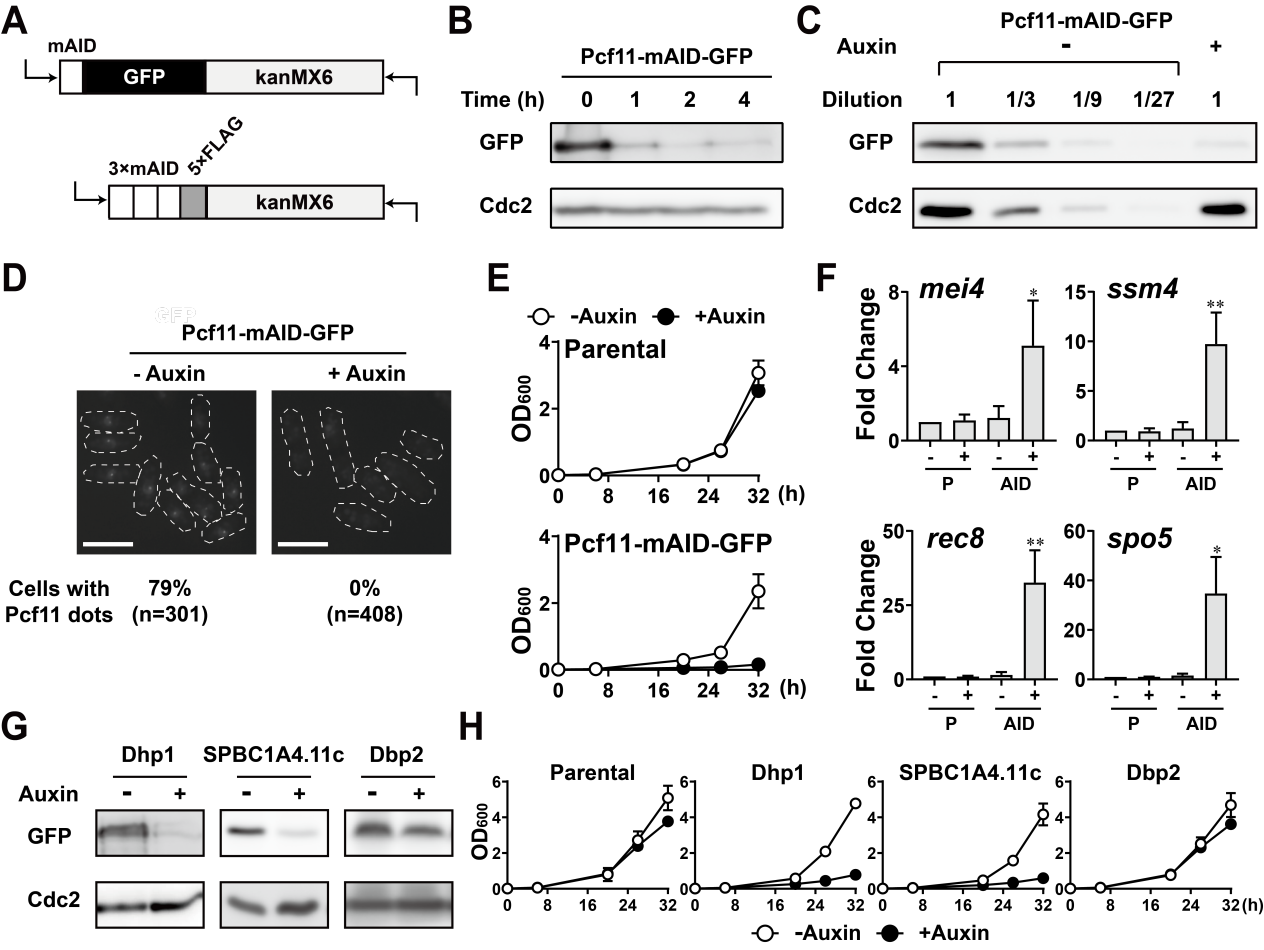
A) Schematic illustration of the mAID-GFP-kanMX6 and 3×mAID-5×FLAG-kanMX6 modules used as PCR templates. B) Western blotting of the Pcf11-mAID-GFP protein using an anti-GFP antibody before (0 hours) and 1, 2, or 4 hours after 1 mM auxin treatment. Western blotting of Cdc2 using the same blot serves as the loading control. C) Western blotting of the Pcf11-mAID-GFP protein using an anti-GFP antibody in the presence (+: 1 mM, 2 hours) or absence (-) of auxin. Three-fold serial dilutions of auxin-absent samples were used to estimate the Pcf11-mAID-GFP level after treatment. Western blotting of Cdc2 serves as the loading control. D) Fluorescence microscopy of Pcf11-mAID-GFP in the presence (1 mM, 2 hours) or absence of auxin*.* Bars, 10 µm. E) Growth curves of Pcf11-mAID-GFP and its parental strains with (1 mM) or without auxin. The mean OD_600_ and standard deviations from three independent experiments were plotted. F) Relative expression of four meiotic mRNAs that are degraded by Red1 in Pcf11-mAID-GFP expressing cells (AID) and its parental cells (P) with (1 mM, 2 hours) or without auxin. The values indicate the means and standard deviations from three independent experiments. The statistical analyses were performed using Student’s *t*-tests: *p<0.05 and **p<0.01. G) Western blotting of Dhp1, SPBC1A4.11c, and Dbp2 fused to 3×mAID-5×FLAG in the presence (1 mM, 2 hours) or absence of auxin. H) Growth curves of 3×mAID-5×FLAG-tagged Dhp1, SPBC1A4.11c, and Dbp2 and its parental strains with or without auxin. The means and standard deviations from three independent experiments were plotted.

## Description

Recent application of the plant auxin-inducible degron (AID) system to the fission yeast *Schizosaccharomyces pombe* added a new option to eliminate a specific protein (Kanke *et al.* 2011). In this system, a protein fused with AID is subjected to proteasome-dependent protein degradation in the presence of auxin and the auxin receptor F-box protein TRANSPORT INHIBITOR RESPONSE1 (TIR1). However, no plasmid was currently available for AID tagging by the PCR-based method (Bähler *et al.* 1998). To expedite the construction of AID-tagged yeast fission strains, we added new assortments of AID-tagging plasmids to the PCR-based gene tagging platform described previously (Bähler *et al.* 1998). When we designed our plasmids for C-terminal AID tagging, we considered the following four criteria. First, the mini-AID (mAID) tag described in the previous study (Nishimura and Kanemaki 2014) rather than the full-length AID (Kanke *et al.* 2011)should be used to minimize the size of the AID tag. Second, a 5×FLAG tag or GFP should be included for downstream experiments such as western blotting and fluorescence microscopy. Third, primer sequences for module amplification should be the same as those described previously to maximize the universality and utility of the genetic components (Bähler *et al.* 1998). Finally, the drug-resistant marker for selection is *kanMX6*, which is one of the most commonly used selection markers for fission yeast. Thus, we constructed two plasmids for use as PCR templates, pFA6a-mAID-GFP-kanMX6 and pFA6a-3×mAID-5×FLAG-kanMX6 (Figure 1A).

To verify the mAID-GFP tag, we constructed a strain expressing Pcf11-mAID-GFP. Pcf11 is an essential component of the pre-mRNA 3´ processing machinery (Larochelle, Hunyadkürti, and Bachand 2017; Liu *et al.* 2017) and colocalizes with Red1, Rhn1, and Erh1, all of which contribute to the suppression of meiotic mRNAs during vegetative growth (Sugiyama and Sugioka-Sugiyama 2011; Sugiyama *et al.* 2012; Sugiyama *et al.* 2016). Upon auxin (1-Naphthaleneacetic acid, 1-NAA) treatment, the Pcf11-mAID-GFP protein level decreased, and the Pcf11-mAID-GFP protein was efficiently depleted within 2 hours (Figure 1B). Serial dilutions of the protein extracts roughly indicated that the level of the Pcf11 protein was reduced to around 1/9 of its original content after the 2-hour auxin treatment (Figure 1C). Consistent with the western blotting results, we did not observe any GFP fluorescence signal in the Pcf11-mAID-GFP cells treated with auxin for 2 hours (Figure 1D). Since Pcf11 is an essential protein (Kim *et al.* 2010; Hayles *et al.* 2013), Pcf11 depletion should result in a growth defect. As expected, Pcf11 depletion adversely affected cell proliferation, whereas auxin treatment did not significantly prevent cell growth (Figure 1E). Besides, four meiotic mRNAs, which are degraded by Red1 during vegetative growth, were enriched in Pcf11-depleted cells (Figure 1F). These data indicated that the mAID-GFP tag interferes with Pcf11 function by reducing the Pcf11 protein level upon auxin treatment.

We next examined whether the 3×mAID-5×FLAG tag could also be used to study essential genes. We chose the 5´ to 3´ exonuclease Dhp1 (Shobuike *et al.* 2001), the RNA helicase Dbp2 (Kilchert *et al.* 2015), and one uncharacterized protein, SPBC1A4.11c, as target proteins. We observed that the levels of Dhp1 and SPBC1A4.11c decreased in response to auxin, but the level of Dbp2 did not obviously change (Figure 1G). Consistent with these western blotting results, Dhp1-3×mAID-5×FLAG and SPBC1A4.11c-3×mAID-5×FLAG yeast lines, but not the Dbp2-3×mAID-5×FLAG line, showed growth retardation in the presence of auxin (Figure 1H).

To further verify the efficacy of our plasmids in the AID system, we also tested whether 3×mAID-5×FLAG tag could work for the RNA helicase Dbp5 and several kinetochore proteins (Mis6, Mis16, Mis18, Scm3, and Sim4). We observed a reduction in the steady-state levels of Dbp5, Mis6, Mis18, and Sim4, but not Mis16 and Scm3 (Song and Xu, data not shown). These results indicate that the 3×mAID-5×FLAG tag and the mAID-GFP tag induced specific protein degradation upon auxin treatment, but AID does not necessarily work for any protein.

There are at least two possibilities for the inefficient proteolysis by mAID. One possibility is that mAID is not accessible to the TIR1-containing E3 ligases depending on its fusion partner. This issue might be fixed by adding tandem mAIDs (e.g., 5×mAID) or swapping the order of mAID and the tags (e.g., 5×FLAG-3×mAID). These changes would help expose mAID to the E3 ligases. The other possibility is that the activity of the two plant TIR1-containing E3 ligases is not optimal in *S. pombe*. A previous study improved the E3 ligase activity by fusing the *S. pombe* Skp1, which is a component of an Skp1-Cul1-F-box protein (SCF) E3 ligase, and the SV40 NLS to TIR1 (Kanke *et al.* 2011). However, there may be room for further optimization of this system, such as by tuning expression levels and incorporating different E3 ligases. Indeed, a preprint describing an improved AID system has been recently published (Zhang *et al.* 2021). Specifically, Zhang *et al.* generated a mutated OsTIR1, OsTIR1-F74A, whose expression is driven by the *adh1* promoter. The OsTIR1-F74A-containing E3 ligase complex efficiently ubiquitinates AID-tagged protein in the presence of the auxin analog 5-adamantyl-indol-3-acetic acid (5-adamantyl-IAA), which can induce protein degradation at a very low concentration (~1 nM) and does not cause auxin toxicity on yeast growth. Since our two plasmids are likely compatible with OsTIR1-F74A, AID tagging using our plasmids should be implemented using the *S. pombe* strain carrying OsTIR1-F74A. We may be able to observe efficient proteolysis, even for Dbp2, Mis16, and Scm3.

Taken together, we have verified two plasmids for the auxin-inducible degron tagging in *S. pombe*. Currently, isolating temperature/cold-sensitive (*ts*/*cs*) mutants is common to study essential genes, and such mutants have been used for many years. However, we like to emphasize two advantages of using mAID (or another degron system) over isolating such mutants. First, constructing mAID-tagged strains using our plasmids is straightforward and time-saving compared to obtaining *ts*/*cs* mutants (Tang *et al.* 2011). Second, auxin-directed proteolysis can be easily prevented and well-regulated by growing mAID-tagged strains without auxin at a non-optimal temperature (at 32°C) for the AID system. Therefore, it does not seem that AID-tagged strains acquire any suppressor mutation(s) readily because AID-tagged proteins confer their normal functions under these conditions. In contrast, *ts*/*cs* mutants can spontaneously acquire a suppressor mutation(s) because such mutations could cause the partial loss of function even under a permissive temperature. Thus, we expect that our plasmids facilitate the study of essential genes in *S. pombe* using the AID system.

## Methods

**Plasmid construction:** The DNA sequence of mAID-GFP-kanMX6 or 3×mAID-5×FLAG-kanMX6 carrying the common primer annealing sequences (forward: 5´-CGGATCCCCGGGTTAATTAA-3´ and reverse: 5´-GAATTCGAGCTCGTTTAAAC-3´) was synthesized and subcloned into the pUC57 vector by Genescript. The resultant plasmids were named pFA6a-mAID-GFP-kanMX6 and pFA6a-3×mAID-5×FLAG-kanMX6, respectively.

**Yeast strain construction and culture:** The fission yeast strain carrying two TIR1 proteins was provided by the NBRP/YGRC of Japan. The C-terminal mAID tagging strains were constructed using the PCR-based method as described previously (Bähler *et al.* 1998). All strains were grown in YES medium (0.5% yeast extract, 3% glucose, and 75 mg/L each of adenine, histidine, leucine, and uracil) at 26°C. For the growth curve, cell cultures in log-phase growth were diluted to the same OD_600_ (0.01) in 6 mL of YES media, and the OD_600_ was measured at the corresponding time points. The concentration of auxin (1-Naphthaleneacetic acid sodium salt, 1-NAASS, Adamas Reagent, Ltd.) was 1 mM.

**Western blotting:** Protein sample preparations for western blotting were performed as previously described (Matsuo *et al.* 2006). Briefly, alkaline-precipitated proteins were resuspended in SDS-sample buffer (60 mM Tris-HCl (pH 6.8), 4% β-mercaptoethanol, 4% SDS, 0.01% bromophenol blue, and 5% glycerol) and were resolved on 10 or 12% SDS-PAGE gels. Anti-GFP (clone 7.1 and 13.1, Roche), anti-DYKDDDDK (HT201-01, Transgene), and anti-Cdc2 (Y100.4, Santa Cruz) antibodies were used for probing the epitope-tagged proteins and Cdc2, respectively. Western blotting was independently done twice, and the representative images are shown in Figure 1.

**Microscopy:** A Zeiss Axio Imager Z2 Upright Microscope (Carl Zeiss MicroImaging) was used for fluorescence microscopy and differential interference contrast (DIC) imaging. The raw images were processed using ZEN lite 2012 (Carl Zeiss MicroImaging).

**RNA analyses:** Yeast cell cultures in log-phase growth were diluted to OD_600 _= 0.6 and then cultured with or without 1 mM 1-NAA for 2 hours. Total RNA was prepared using a MasterPure™ Yeast RNA Purification Kit (Lucigen). RT-qPCR was performed using the PrimeScript^TM^ II 1st strand cDNA synthesis kit (TaKaRa Bio Inc.) and ChamQ^TM ^Universal SYBR^®^ qPCR Master Mix (Vazyme). The RT-PCR analyses were independently performed three times according to the manufacturer’s protocol. The *act1* mRNA was used to normalize the expression levels of the four meiotic mRNAs (*mei4*, *rec8*, *ssm4*, and *spo5*), and the fold increases were calculated and shown. DNA contamination in RNA samples was tested using RNA lacking reverse-transcription reaction components. The *act1* mRNA Ct values of samples with reverse transcription components were approximately 18, whereas those of reverse transcription minus samples were more than 34. Therefore, we concluded that residual DNA in our RNA samples is, if present, negligible.

## Reagents

**Table:** 

Name	Genotype	Used in	Source
FY21104	*h^–^ ura4D18 ade6::ade6^+^-Padh15-skp1-AtTIR1-2NLS Padh15-skp1-OsTIR1*	E,F, and H	NBRP/YGRC
SHP62	*h^–^ ura4D18 ade6::ade6^+^-Padh15-skp1-AtTIR1-2NLS Padh15-skp1-OsTIR1* *SPBC1A4.11c-3×mAID-5×FLAG-kanMX6*	G and H	this study
SHP166	*h^–^ ura4D18 ade6::ade6^+^-Padh15-skp1-AtTIR1-2NLS Padh15-skp1-OsTIR1* *pcf11-mAID-GFP-kanMX6*	B,C,D,E, and F	this study
SHP318	*h^–^ ura4D18 ade6::ade6^+^-Padh15-skp1-AtTIR1-2NLS Padh15-skp1-OsTIR1* *dbp2-3×mAID-5×FLAG-kanMX6*	G and H	this study
SHP526	*h^–^ ura4D18 ade6::ade6^+^-Padh15-skp1-AtTIR1-2NLS Padh15-skp1-OsTIR1* *dhp1-3×mAID-5×FLAG-kanMX6*	G and H	this study

**Table:** 

Primer name	Sequence (5´ to 3´)
mei4 121 Fw	CGACGCGAGAGATACCATTAG
mei4 121 Rv	GGTTGACTTGCATCGTTTGAG
ssm4 142 Fw	TGTACCGGGAAGTTTGGATTTA
ssm4 142 Rv	CAACAGTTGCCTTCTTGTCTTC
rec8 979 Fw	CGTAGAGGAGCTTCTTCGGC
rec8 979 Rv	TCAAGCAAGGGGGTGGAATG
spo5 1231 Fw	TCAAACCGCGTACTTCGTGA
spo5 1231 Rv	TGTGTTCGACTCCAATGGCA
act1 Fw	CCCAAATCCAACCGTGAGAAGATG
act1 Rv	CCAGAGTCCAAGACGATACCAGTG
